# Circular RNAs modulate Hippo-YAP signaling: functional mechanisms in cancer

**DOI:** 10.7150/thno.71708

**Published:** 2022-05-16

**Authors:** Javeria Qadir, Feiya Li, Burton B. Yang

**Affiliations:** 1 Sunnybrook Research Institute, Sunnybrook Health Sciences Centre, Toronto, Canada; 2 Department of Biosciences, COMSATS University Islamabad, Islamabad, Pakistan; 3 Department of Laboratory Medicine and Pathobiology, University of Toronto, Toronto, Canada

**Keywords:** Hippo pathway, YAP, circular RNA, circRNA, signal transduction, cancer progression

## Abstract

The Hippo signaling pathway is an evolutionarily conserved network that regulates organ size and tissue homeostasis in mammals. This pathway controls various cell functions, such as growth, proliferation, survival, apoptosis, and stemness by switching 'on' or 'off' its inhibitory and/or transcriptional module, thereby regulating target gene(s) expression. Altered Hippo signaling has been implicated in various forms of cancers. Increasing evidence suggests cross-talk between the Hippo signaling pathway and non-coding RNAs, in particular circular RNAs (circRNAs). In this context, the current review presents the mechanistic interplay between the Hippo pathway and related circRNAs in various forms of cancers, along with the capabilities of these circRNAs to function either as tumor suppressors or oncogenes through miRNA sponging or protein binding mechanisms. Furthermore, we discuss the constraints and limitations in circRNA mechanistic studies while highlighting some outstanding questions regarding the roles of circRNAs associated with the Hippo-YAP pathway in cancer. Finally, we delineate the potential of these circRNAs to be employed as diagnostic and prognostic biomarkers, as well as molecular hotspots for cancer therapy.

## 1. Introduction

An evolutionary perspective has shown organ size control as a principal inquisition in the domain of biology since its inception. Despite the substantial research carried out so far, our understanding about how organ sizes are maintained through the course of human development is still fragmented. Progress in addressing this enigma has been made since the discovery of the role of the Hippo pathway in control of organ size, primary cellular processes and auxiliary regulatory mechanisms 1, 2.

**Figure 1:**
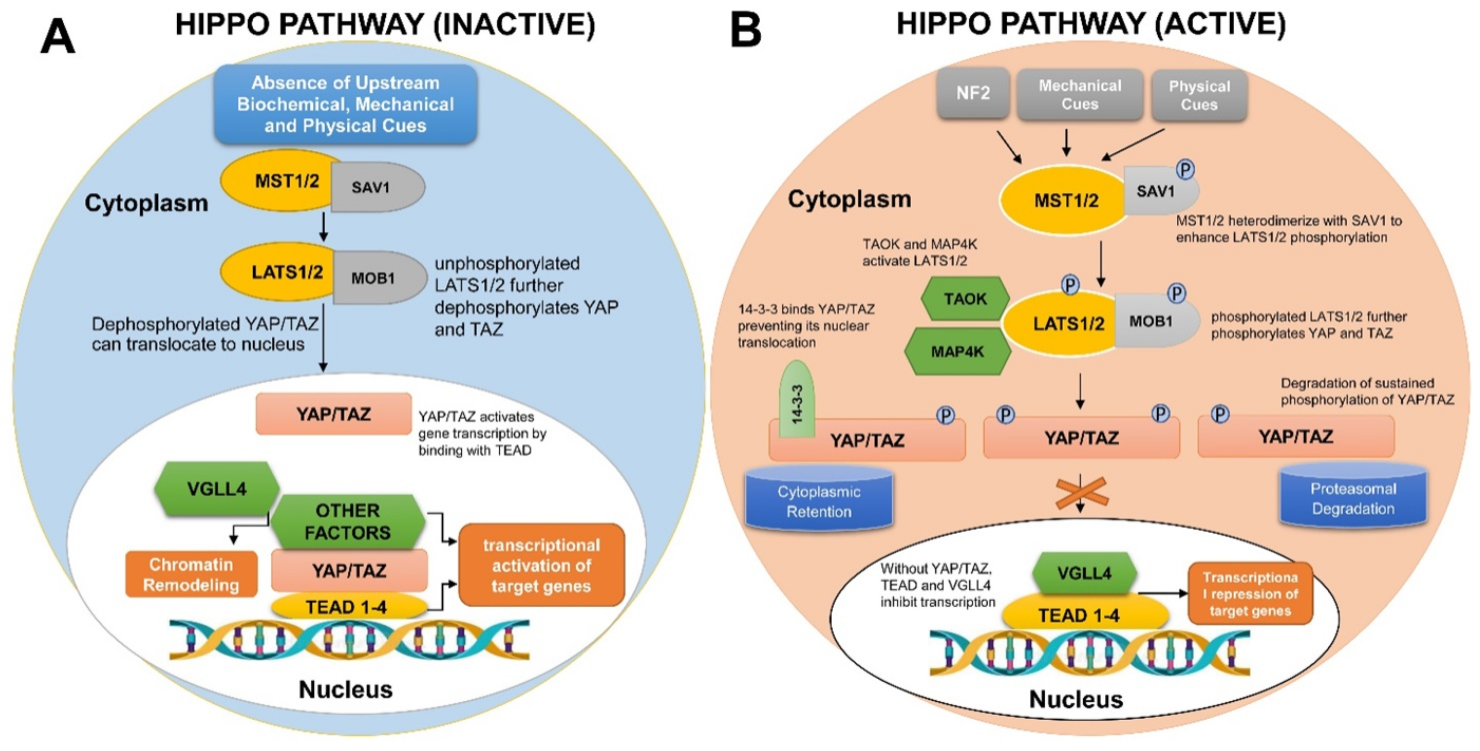
**Mechanistic details of Hippo-YAP signaling pathway in human cells. (A)** If the Hippo pathway is switched off or inactivated, YAP/TAZ remain unphosphorylated and are translocated into the nucleus to bind TEAD group of transcription factors, therefore, regulating the transcription of gene(s) necessitated for cell proliferation and survival. **(B)** Upon activation of Hippo signaling pathway, numerous biochemical, mechanical and physical cues bring about MST1/2 and LATS1/2 phosphorylation. Phosphorylated LATS1/2 Kinases further phosphorylate YAP/TAZ, therefore, engaging either 14-3-3 or Ubiquitin proteins to mediate YAP/TAZ cytoplasmic retention or ubiquitylation/ proteasomal degradation, respectively.

Contrary to the conventional ligand-receptor binding observed in many cellular signaling pathways, the Hippo pathway is triggered in response to a wide variety of physical, mechanical and biochemical signals, thus regulating diverse biological processes. First discovered in *Drosophila melanogaster*, the Hippo pathway is an evolutionary conserved network that regulates organ sizes and tissue homeostasis in mammals 3, 4. It is regarded as the master regulator of the developmental events that occur during the course of organogenesis, tissue regeneration, and tumorigenesis 5, 6. The core molecular components constituting the pathway along with the pathway activation and inactivation processes are depicted in Figure 1.

Non-coding RNAs, including microRNA (miRNA), long-chain non-coding RNA (lncRNA), small interference RNA (siRNA), and circular RNA (circRNA) are known to make up more than 75% of the genome transcripts and regulate various physiological processes during development and tumorigenesis 7, 8. These regulatory functions are brought about through the interaction of these non-coding RNAs with several other signaling pathways 9, 10. In the past decade, convincing evidence has accumulated in regards to the roles of non-coding RNAs, in particular circRNAs, in a wide range of physiological processes, as well as the pathogenesis of various diseases including cancer 11.

Circular RNAs are largely pre-mRNAs derivatives that are formed as a result of back splicing, unlike linear RNAs that follow the standard canonical splicing process 12, 13. Owing to their covalently closed structure and restricted degradation by exonucleases, circRNAs have been observed to be more stable in comparison to their linear counterparts 14. Generally, circRNA biogenesis is either mediated through alternative back-splicing 13, lariat-driven mechanisms 15, intron pairing-based circularization 16, or circularization that follows pairing with RBPs (RNA Binding Proteins) 17, 18.

Circular RNAs have been shown to impart functions by regulating various signaling cascades during tumorigenesis 19, 20. Interestingly, molecular interaction between circRNAs and the Hippo pathway is highly intricate, which requires intense research studies to be performed. Hippo pathway components have also been inadequately reported to be involved in circRNA-mediated regulatory mechanisms. In the current review, the plausible molecular interactions between circRNAs and the Hippo pathway components along with their functional implications in the process of carcinogenesis have been comprehensively outlined. Furthermore, circRNA-mediated regulation of the Hippo-YAP signaling axis with significant implications in devising therapeutic regimens for patients with various forms of cancer is discussed.

## 2. Regulation of Hippo-YAP Signaling Axis by circRNAs

Circular RNAs are single-stranded circularized RNA molecules that are generated from linear mRNA as a consequence of back-splicing 21, 22. Until recently, circRNAs were considered to be products of anomalous RNA splicing 23, 24. Now we know that circRNAs are prevalent and abundantly expressed in various tissues. They are rather more stable in comparison with their linear counterparts due to their resistance to ribonuclease (RNase) activities 25. Circular RNAs have been proposed to function by means of miRNA sponging 26, 27, regulating transcription 28, 29 and interacting with RNA-binding proteins (RBPs) 14, 18, 30, 31. Genome wide analysis has also demonstrated that circRNAs are evolutionary conserved among species 32, 33. In the field of cancer biology, circRNAs have been found to significantly impact tumor cell growth 34-36, proliferation 37, 38, invasion 39-41, adhesion 42, 43, metastasis 44-47 and apoptosis 48, 49, suggesting their potential to be used as diagnostic and prognostic biomarkers, as well as therapeutic agents 50-52.

Likewise, these circularized transcripts have also been found to reciprocally interact with the Hippo-YAP signaling pathway, especially during cancer development and progression 53, 54. Primary components of the Hippo pathway are broadly classified into two distinct modules; (i) Regulatory (inhibitory) module and (ii) Transcriptional module 55-57. In this section, circRNAs that interact with these two modules of the Hippo signaling pathway in various forms of human cancer are discussed in detail.

### 2.1. Circular RNAs Associated with the Regulatory (Inhibitory) Module

The regulatory module of the Hippo signaling pathway consists of kinases, including sterile 20 like kinase 1 and 2 (MST1/ MST2), Large Tumor Suppressor 1 and 2 (LATS1/ LATS2), Salvador homolog 1 (SAV1) and MOB Kinase activator 1A and 1B (MOB1A/ MOB1B) 58, 59. The switching “ON” and “OFF” of the Hippo signaling cascade is largely controlled by this series of core kinases as illustrated in Figure 1. Even though there is an influx of studies on the roles of circRNAs in cancer, only a few reports focus on Hippo pathway regulation through circRNAs and their roles in cancer progression.

**Figure 2:**
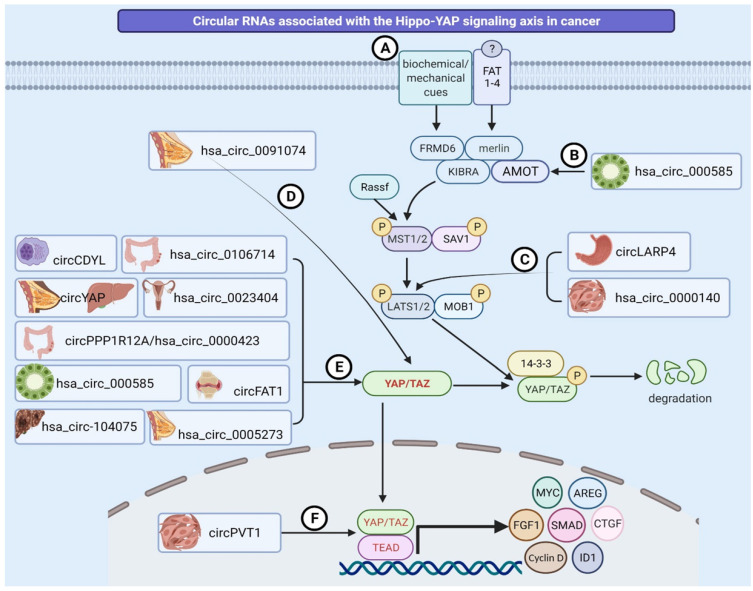
** Diagrammatic illustration of circRNAs regulating the Hippo-YAP signaling pathway. (A)** The Hippo pathway is triggered in response to a wide variety of physical, mechanical and biochemical signals, or by FAT family of Cadherins (FAT1-4), thus regulating diverse biological processes and pathological states including cancer. **(B)** hsa_circ_000585 binds to miR-615-5p and modulates AMOT activity in cholangiosarcoma. **(C)** circLARP4 and circRNA_0000140 regulate LATS1/2 expression by sponging miR-424-5p and miR-31 in gastric cancer and oral squamous cell carcinoma, respectively.** (D)** Hsa_circ0091074 controls TAZ activity by forming an endogenous sponge for miR-1297.** (E)** Hsa_circ_0023404, circPPP1R12A, circRNA_104075, circDDYL, circFAT1, circ0106714, circRNA_000585, and hsa_circ_0005273 act as sponges for miRNAs in cervical cancer, colon cancer, hepatocellular carcinoma, multiple myeloma, osteosarcoma, colorectal cancer, cholangiosarcoma, and breast cancer, respectively. In contrast, circYAP inhibits YAP1 translation through protein binding mechanisms and acts as tumor suppressor in breast and liver cancers. **(F)** circPVT1 binds to miR-497-5p and alters the activity of the YAP/TEAD complex in head and neck squamous cell carcinoma.

Circular RNAs are known to impart either tumor suppressive 60 or oncogenic functions in several types of cancers 61-63. However, there are only two comprehensive studies that discuss the cross-talk between regulatory (inhibitory) module of the pathway and circRNAs in cancer. One of the studies reported the tumor suppressive function of circLAPR4 in gastric cancer. It was observed that circLARP4 sponges miR-424-5p, therefore suppressing tumor cell proliferation and invasion in a LATS1-dependent manner 64. Similarly, circRNA_0000140 has also been noted to regulate LATS2 expression through miR-31 sponging mechanism, thus acting as tumor suppressor in oral squamous cell carcinoma 65 (Table 1, Figure 2).

### 2.2. Circular RNAs Associated with the Transcriptional Module

The transcriptional module is composed of two closely associated molecular entities i.e., transcriptional co-activator (TAZ) and Yes associated protein (YAP) 66-70. As YAP/TAZ complex subsequently acts as a downstream effector, it interacts with a range of sequence specific transcription factors TEAD1-4 56, 71. Many studies in this review spotlight the regulation of the Hippo-YAP transcriptional machinery by various circRNAs in cancer (Figure 2, Table 1). Given that the primary effects of the Hippo pathway are produced through YAP/TAZ and the TEAD transcription complex, most of the studies discussed in this review pertain to circRNAs that regulate the transcriptional module of the Hippo signaling pathway. Only a small number of studies support the significance of circRNAs in regulating inhibitory module 64, 65.

In cervical cancer, hsa_circ_0023404 has been reported to play an oncogenic role by sponging miR-136 and subsequently modulating YAP1 expression 72. Moreover, circPPP1R12A (hsa_circ_0000423) has been elucidated to potentiate tumor progression and metastatic spread by activating the Hippo-YAP signaling pathway, acting as an oncogene and poor prognostic marker in colon cancer 73. Similarly, circRNA_104075, circ-CDYL, circRNA_000585, hsa_circ_0005273, and circFAT1 have also been reported to have oncogenic functions by modulating YAP1 activity through miRNA binding in hepatocellular carcinoma 74, multiple myeloma 75, cholangiosarcoma 76, breast cancer 77, and osteosarcoma 78, respectively. Conversely, hsa_circ_0106714 was also noted to alter YAP1 expression by sponging miR-942-5p, acting as a tumor suppressor in colorectal cancer 79. Interestingly, our lab uncovered a tumor suppressive function of circYAP in breast cancer, liver cancer and mouse melanoma cells 80. This is the first and only report to reveal a protein binding mechanism of circRNA function in this pathway.

Furthermore, circPVT1 was found to be upregulated in head and neck squamous cell carcinoma. It was also observed to function through miR-497-5p sponging mechanism by targeting YAP/TEAD transcription complex 81. In addition to YAP1, TAZ is an important downstream effector of the Hippo pathway that serves as an integral component of its transcriptional module. It acts as a transcriptional co-activator alongside YAP1 to bring about target gene(s) transcription in response to pathway activation 66. Thus far, there are only two research studies that describe the modulation of TAZ by circRNAs in breast cancer. In addition, hsa_circ_0091074 has been shown to act as an endogenous sponge for miR-1297 and serve as a potential therapeutic target for triple negative breast cancer subtype 82. Yet another study on breast cancer reported an oncogenic function of circ_0001667 by regulating TAZ expression. The study revealed circ_0001667 to be a potential marker of prognosis, as well as a plausible therapeutic target for metastatic breast cancer by modulating the miR-125a-5p/TAZ axis 83.

## 3. Functional Mechanisms of circRNAs Associated with Hippo-YAP Signaling

To date, circRNAs have been proposed to function through several molecular mechanisms, including (i) miRNA sponging, (ii) mRNA regulation at both translational and transcriptional levels, (iii) interacting with proteins to form circRNA-protein complexes, and (iv) translation into a peptide or a protein 84-86. However, circRNA binding to mRNA and proteins is often taking place at odds and not always concordant. Interestingly, most of the studies involving circRNAs have been in the field of cancer 21, 84, 87. Thus far, only a handful of studies have reported about the circRNAs regulating the Hippo-YAP signaling pathway. Of these, more than 90% of the studies spotlight the miRNA sponging potential of these circRNAs, and only one study documented their protein binding efficiency in association with the Hippo-YAP signaling axis 80. These functional capabilities of circRNAs are reviewed in the subsequent sections.

### 3.1. Protein Binding

Regulation of genes at the translational level is an important aspect of cancer initiation and progression, which typically involves mRNA circularization and binding of RBPs to the translation initiation complex on mRNA 88, 89. Our lab recently discovered the YAP protein to be negatively regulated by circular YAP. circYAP interfered with YAP1 translation initiation machinery assembly, thereby repressing proliferation, colony formation and migration in breast cancer and liver cancer cells. To our surprise, circYAP was found to block YAP1 protein synthesis while its mRNA levels remained intact. However, it was observed that the binding of circYAP to YAP mRNA and the proteins eIF4G and PABP associated with translation initiation, formed a quaternary complex that abolished the initiation of YAP translation 80.

### 3.2. miRNA Sponging

MicroRNAs (miRNAs) are small noncoding RNAs that are usually 22 nucleotides in length. In the cytoplasm, these small non-coding RNAs bind to target mRNAs and induce post-transcriptional silencing 90. miRNAs have crucial functions during physiological as well as pathological processes 91. In 2013, it was demonstrated that circRNAs could act as “miRNA sponges” by binding and sequestering miRNA away from their target mRNA, thereby weakening miRNA-mediated gene suppression 92. Following this study, more and more circRNAs were found to act as miRNA sponges 93. In the cancer field, this sponge mechanism has been implicated in cell proliferation 94, 95, migration 96, and angiogenesis 97.

Due to the advantages of RNA-sequencing techniques and the advancements of new computational schemes specifically designed for circRNA analysis, increasing studies have screened out circRNA candidates that are potentially functional, and more experiments are in the pipeline to elucidate the roles of circRNAs in various pathologies, including cancer 98, 99. Most of the circRNA studies have been focused on the cancer field. However, based on RNA sequencing technologies, the major limitations that come up with targeting circRNAs is their off-target gene(s) silencing and/or non-specific cell-or tissue-type targeting. Sequence similarity between circRNA and its cognate linear mRNA limits the usefulness of RNA Seq for screening target circRNAs. Additionally, these techniques have been found to demonstrate reduced sensitivity in detecting circRNAs that are expressed at relatively low or moderate levels. Therefore, more subtle techniques and target enrichment approaches to enable screening of circRNAs with low or moderate expression are required.

**Figure 3:**
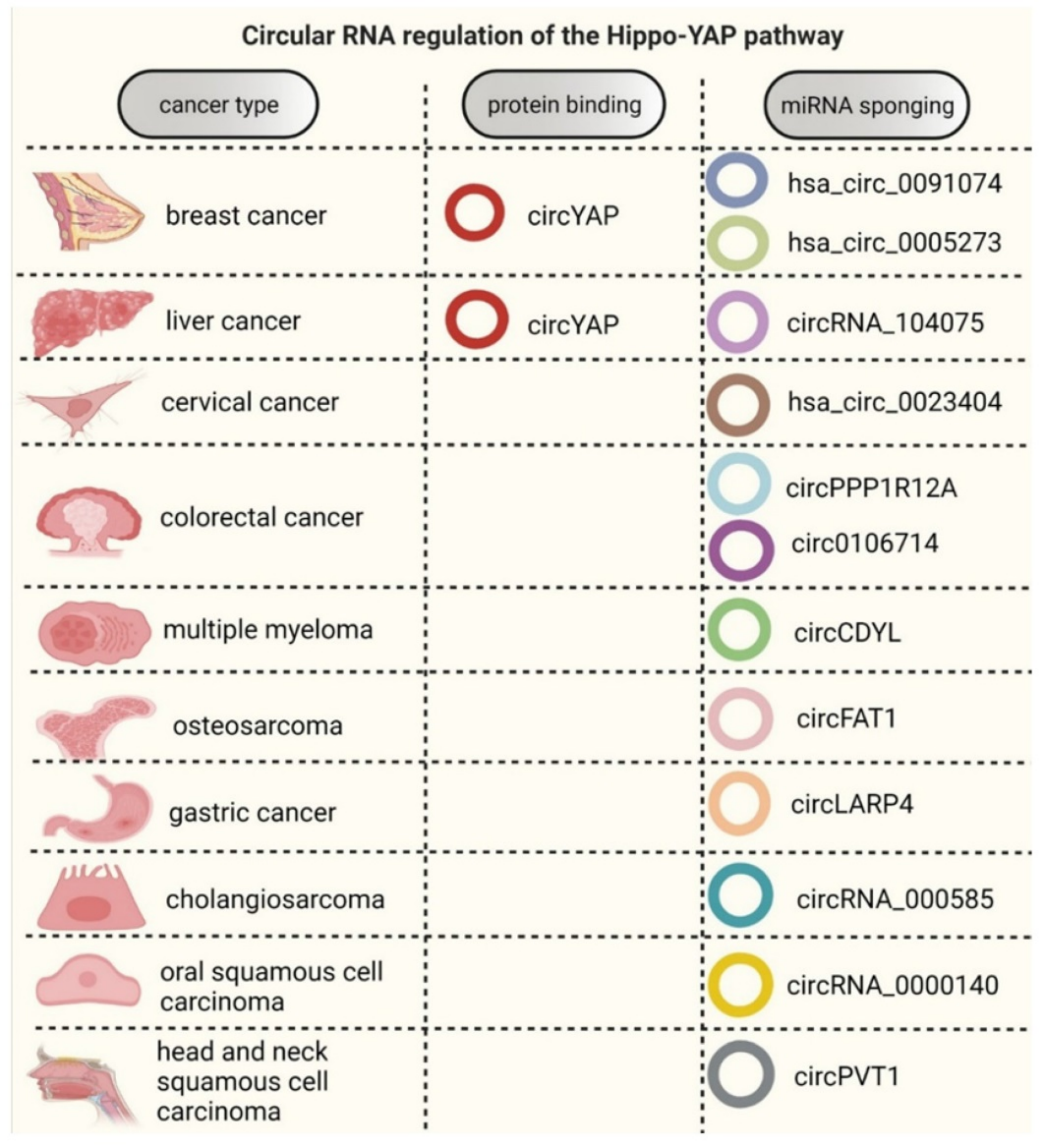
** Functional mechanisms of circRNAs modulating the Hippo-YAP signaling pathway in various forms of cancer.** This figure illustrates the circRNAs associated with the Hippo-YAP signaling axis in different forms of human cancers. The left column indicates the types of cancer in which these related circRNAs are functionally implicated. The column in the middle depicts the circRNA that has been described to alter YAP1 expression in breast cancer and liver cancer through an efficient protein binding mechanism that halts YAP1 translation. The right column lists those circRNAs that have been observed to act as sponges for miRNA, leading to cancer development and progression.

During the past decade, there has been a surge in circRNA-based studies, however, only a limited number of studies reported the cross-talk between the Hippo-YAP signaling pathway and circRNAs in the domain of cancer. Out of these, more than 90% of studies have proposed miRNA sponging for Hippo-YAP regulation by circRNAs in cancer (Table 1, Figure 3). Owing to their cell/tissue-type specific expression and functions, a circRNA may be abundantly expressed in one tissue type while displaying very low levels in another. Similarly, a circRNA may have pathological functions in one tissue type and may retain its physiological roles in another tissue. Likewise, a particular circRNA acting as a miRNA sponge in one form of cancer may not necessarily act as miRNA sponge in all other cancer types. Therefore, increasing evidence on their tissue specific expression may not seemingly strengthen the pan-carcinogenic value of these circRNAs. The circRNAs that have been reported to possess sponging capabilities for miRNAs in interacting with Hippo-YAP pathway molecules in various forms of cancers are discussed below.

#### 3.2.1. Cervical Cancer

Dysregulated expression of circRNAs has been systematically explored in cervical cancer 100, 101, which is one of the most malignant forms of cancer in females 102. Multiple studies have also highlighted the roles of these circRNAs as potential diagnostic markers in cervical cancer 103, 104. circRNA hsa_circ_0023404 has been reported to mediate tumor formation. This circRNA was found to be overexpressed in the diseased tissues when compared to their respective controls and was poorly associated with survival in these patients. Functional studies revealed a decline in cell proliferation, migration and invasion of the cancer cells upon hsa_circ_0023404 knockdown. It was reported to hold a miRNA sponging mechanism by binding to miR-136 and subsequently altering YAP1 expression and its activity in cervical cancer 72.

#### 3.2.2. Colon/ Colorectal Cancer

Current high-throughput approaches have unveiled a number of circRNAs that are dysregulated in colon cancer tissues 105. These circRNAs have also been implicated in various pathological states, including colon/colorectal cancer 87. Two studies reported dysregulation of YAP1 expression in colorectal cancer as a consequence of circPPP1R12A (hsa_circ_0000423) and circ0106714 binding with the target miRNA. circPPP1R12A was found to have an oncogenic function 73, 106, whereas circ0106714 showed tumor suppressive function as its upregulation reverted the malignant phenotype of colon tumor cells through miR‐942‐5p sponging mechanisms that led to enhanced YAP1 phosphorylation 79. On the contrary, circPPP1R12A was upregulated in colon cancer tissues in comparison to their respective controls with poor survival outcomes. It also increased tumor characteristics like proliferation, survival, invasion, growth and metastasis in both *in vitro* as well as *in vivo* studies 106.

#### 3.2.3. Hepatocellular Carcinoma

Differential expression of various circRNAs has been investigated in various forms of liver related pathologies 107, including liver cancer 108, 109. In regards to circRNA regulation, some circRNAs have been noted to be dysregulated in hepatocellular carcinoma (HCC) 110, 111. However, only one study documented overexpression and a role of circRNA_104075 in regulating YAP1 expression in HCC by acting as ceRNA through miR-582-3p binding. It was found to have diagnostic potential and could possibly serve as a therapeutic target for HCC clinical management 74.

#### 3.2.4. Multiple Myeloma

Heterogeneous expression of circRNAs has been explored in multiple myeloma and associated with their diagnostic as well as prognostic potential 112. A study revealed the role of a circRNA named circCDYL in multiple myeloma. It was found to be increasingly expressed in tissue as well as plasma samples with a good prognostic value. circCDYL has been observed to reduce apoptosis and increase tumor cell viability by miR-1180 binding, followed by an increase in YAP1 expression. *In vivo* functional studies also validated the regulatory function of circCDYL/miR-1180/YAP axis and its potential to be exploited as a therapeutic target in multiple myeloma patients 75.

#### 3.2.5. Osteosarcoma

Osteogenic differentiation of bone marrow stem cells has been revealed to involve differential expression of circRNAs 113. Also, these circRNAs have been implicated in various stages of bone development and bone related diseases in humans 114, 115. circFAT1 was found to be upregulated in bone cancer tissues and cells, and was shown to have oncogenic activity in the studied specimens. Both *in vitro* and *in vivo* study models showed a decrease in tumor characteristics, including tumor growth, invasion, and migration. circFAT1 was examined to bind to miR-375, acting as a miRNA sponge to modulate YAP1 expression. Therefore, circFAT1 could potentially be used as a therapeutic target for osteosarcoma 78.

#### 3.2.6. Head and Neck Squamous Cell Carcinoma

Circular RNAs have been shown to exert functions during tumor growth, proliferation, invasion, migration, and chemo-sensitivity in Head and Neck Squamous Cell Carcinoma (HNSCC) 116, 117. Increased circPVT1 expression has been found to modulate activity of YAP/TEAD complex in HNSCC, particularly in patients exhibiting TP53 mutations. Moreover, circPVT1 upregulation is also reported to result in a more malignant phenotype, thus acting as an oncogene by sponging with miR-497-5p 81.

#### 3.2.7. Cholangiosarcoma

Thus far, a small number of reports addressing expressional dysregulation of various circRNAs in cholangiosarcoma have been published 118-120. Concerning the Hippo signaling pathway, circRNA_000585 has been reported to be upregulated in tumors, alters AMOT and YAP1 expression in cholangiosarcoma through miR-615-5p binding mechanism and may be used as a potential target for treating cholangiosarcoma 76.

#### 3.2.8. Gastric Cancer

Studies have outlined the role of numerous circRNAs in regulation of gastric cancer (GC) 121-123. In regards to the Hippo-YAP pathway, circLARP4 has been observed to sponge miR-424 and exhibit tumor suppressive function in gastric cancer. circLARP4 inhibitory effects were reported to occur through modulation of LATS1 expression, mitigating proliferation, invasion, and acting as a potential marker of poor prognosis in GC 64.

#### 3.2.9. Oral Squamous Cell Carcinoma

Oral squamous cell carcinoma (OSCC) is one of the most prevalent form of Head and Neck Cancer with reduced overall survival across the globe 124. Several studies have identified different circRNAs with functional implications in OSCC 125, 126. circRNA_0000140 has been reported to be dysregulated in both *in vitro* and *in vivo* OSCC models, in which it suppressed proliferation, migration, and invasion while increased apoptosis. Further, circRNA_0000140 was found to carry out these functions by targeting LATS2, a critical component of the inhibitory module of the Hippo signaling pathway, via miR-31 sponging 65.

#### 3.2.10. Breast Cancer

Being the most prevalent form of cancer in women, crucial roles of different circRNAs have been recognized in breast cancer 34, 127-130, and analyzed through microarray and sequencing techniques 131. Of the four intrinsic molecular breast cancer subtypes 132, Triple Negative Breast Cancer (TNBC) is the most aggressive subtype with the highest mortality, increased metastatic spread and limited treatment options 133, 134. Interestingly, a study revealed that the binding of hsa_circ_0091074 with miR‑1297 in breast cancer resulted in upregulation of TAZ expression. Hsa_circ_0091074 was suggested to increase proliferation, invasion, and migration, and hence could be employed as a potential therapeutic target in TNBC 82. Yet another study reported an oncogenic role of hsa_circ_0001667 in breast cancer cells and tissues by acting as an endogenous sponge for miR-125a-5p, thus regulating TAZ expression 83. Additionally, hsa_circ_0005273 was observed to be upregulated in breast cancer cells and tissues and bind to miR-200a-3p to stimulate YAP1 expression, ultimately inactivating the Hippo pathway. Therefore, it can serve as a potential prognostic marker and a therapeutic target in combating breast cancer 77.

## 4. Applications of Hippo-YAP Associated circRNAs in Cancer Theranostics

In the last two decades, a large pool of studies has informed us about the roles of circRNAs in different types of cancers, whereby dysregulated expression of these circRNAs is reported to regulate various molecular entities as well as complex cell signaling cascades 28, 135, 136. So far, several studies have highlighted the critical functions of circRNAs in altering basic tumor characteristics such as growth 137, 138, proliferation, invasion, migration, and metastasis 35, 39. In addition to these, their potential to be used as biomarkers and therapeutic targets have also been extensively elucidated in the field of cancer biology 21, 139. Nevertheless, limited evidence is available in regards to the clinical applications of those circRNAs that specifically regulate Hippo-YAP signaling during cancer progression. The subsequent section discusses the utility of the reviewed circRNAs associated with the Hippo pathway as prognostic biomarkers and/or therapeutic targets.

### 4.1. Circular RNAs as Prognostic/Diagnostic Biomarker

**Figure 4:**
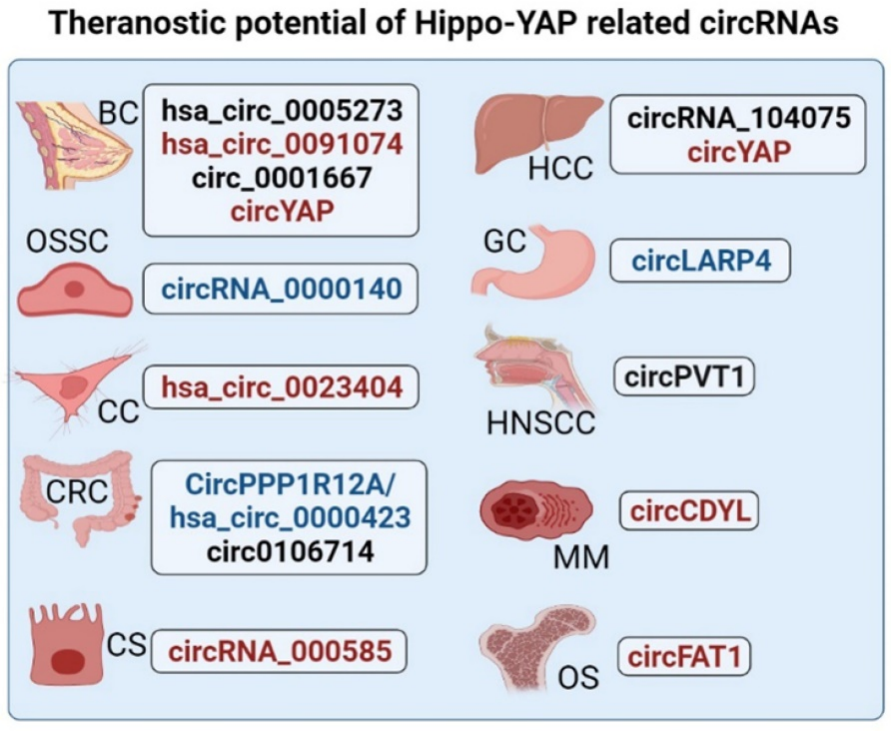
** Potential applications of Hippo-YAP related circRNAs in cancer prognosis/diagnosis and/or therapy.** This figure displays the Hippo-YAP associated circRNAs with prognostic/diagnostic and therapeutic significance in different forms of cancers. circRNAs that can potentially be employed as prognostic/diagnostic markers are colored blue and those reported to have possible therapeutic value are colored red. Whereas, circRNAs marked black are indicative of both prognostic and therapeutic significance in respective cancer type. (Where BC = Breast Cancer, HCC = Hepatocellular Carcinoma, GC = Gastric Cancer, OSSC = Oral Squamous Cell Carcinoma, HNSCC = Head and Neck Squamous Cell Carcinoma, CC = Cervical Cancer, CRC = Colorectal Cancer, OS = Osteosarcoma, MM = Multiple Myeloma, and CS = Cholangiosarcoma).

During the course of cancer development, histopathological characteristics are predominantly altered as a consequence of changes at molecular levels. Although the functional mechanisms of circRNAs in disease pathogenesis are yet to be completely elucidated, their potential to be used as biomarkers for early cancer diagnosis/prognosis cannot be undervalued. Owing to high tissue specificity and sensitivity, circRNAs may be regarded as promising biomarkers for diagnosis and predicting prognosis in cancer patients. Similarly, various studies have revealed the prognostic power of circRNAs associated with Hippo-YAP pathway and their application as biomarkers in different cancer types where the dysregulated circRNAs have been shown to impact survival outcomes in cancer patients, as depicted in Figure 4.

Circular RNAs are resistant to exonuclease digestion owing to the unavailability of 5' and 3' ends in their structure, therefore presenting a longer half-life in comparison to their linear counterparts 23, 25, 140. Since circRNAs can be identified in a variety of biological specimens, including cells 141, plasma 142, 143, serum 144, tissue, urine 43, 145-147, exosomes 148-150, and saliva 151, they could be used as potential cancer biomarkers if dysregulated. Moreover, circRNAs have been detected in different peripheral blood components that include blood cells, extracellular vesicles as well as the plasma 152. These components can be efficiently used as liquid biopsy markers for diagnosing different types of human diseases including cancer 153, 154. Likewise, the circRNAs interplaying with Hippo-YAP signaling may represent plausible diagnostic and prognostic biomarkers. A detailed picture of these circRNAs have been depicted in Table 2 and Table 3.

### 4.2. Circular RNAs as Therapeutic Modulators

For adequate cancer management, the choice of therapeutic regimen primarily relies on the type and/or severity of the cancer. Due to increased diversity, heterogeneity and complex underlying molecular mechanisms, personalized therapies (such as targeted therapies or precision medicine) are essentially needed. Numerous inhibitors targeting YAP, a major oncoprotein of the Hippo pathway, have been described earlier. Nonetheless, whether the Hippo-YAP-interacting circRNAs can serve as potential therapeutic agents or targets is still conditional to the findings of the translational studies in progress, and requires extensive *in vivo* testing for subsequent development of circRNA based therapies.

Since circRNAs present altered expression profiles in different pathological states in humans, their expression levels can help emphasize their value as therapeutic modulators 155-157. Table 2, Table 3 and Figure 4 describe the circRNAs that work through modulation of Hippo-YAP signaling and could have therapeutic applications in cancer. These circRNAs have the potential to be used either as a therapeutic agent or a therapeutic target depending upon the state of their expressional dysregulation i.e., upregulation or downregulation in a particular pathological state. In this regard, Table 4 describes the roles of the studied circRNAs as therapeutic modulators in various cancers. Comprehensively, more subtle techniques and thorough studies, both at molecular and translational levels, are required to establish the clinical significance of circRNAs related to Hippo-YAP pathway, and their roles in incurring resistance to chemotherapy, radiotherapy and endocrine therapies.

## 5. Discussion

Over the span of the last few decades, there have been thorough studies on the Hippo signaling pathway in mammals, specifically in humans. Intense research has generated a large amount of data about the Hippo-YAP axis and its interplay with various other signaling pathways, as well as regulatory molecular entities, including non-coding RNAs. Of these, there is emerging evidence for the mechanistic regulation of the Hippo signaling pathway by a relatively new class of non-coding RNAs, circRNAs, in the sphere of cancer biology. The current review outlined the functional mechanisms of circRNAs associated with the Hippo pathway in cancer and how these circularized transcripts have the potential to regulate various pathway components that constitute its regulatory (inhibitory) and transcriptional modules. Still, there are a multitude of dimensions that have yet to be explored and several important concerns that have yet to be addressed, as discussed below.

### 5.1. miRNA Sponging Mechanism: Constraints and Limitations

First discovered in 2013, hundreds of circRNAs have been profiled for their miRNA sponging capabilities^92^. More than 90% of the studies recruited in this review discussed this sponging mechanism. However, not every sponge being proposed may function as a sponge physiologically. Relevant to this 158, we recently reviewed key considerations in circRNA research, such as the relative abundance of circRNAs to their binding partner (miRNA or RBP), which should be considered to understand their functional mechanisms in cancer.

Concerning circRNA binding to target miRNA, the primary constraint that comes up with this mechanism is lack of copy number measurements for both circRNA and miRNA prior to investigating plausible functions. Being already informed about the higher copy number of miRNAs as compared to the circRNAs, it would be imperative to assess circRNA to miRNA ratio in order to add to the authenticity of miRNA sponging as a potential mechanism for circRNA function. Furthermore, the number of binding sites in the target miRNA must also be taken into consideration to validate miRNA sponging as the proposed mechanism for circRNA activity. This is because if the circRNA expression level is low, it might not be able to occupy all the miRNA binding sites to act as a sponge in true sense. Also, if there exist only few binding sites in the target miRNA for the respective circRNA, the circRNA might still not function as a true sponge. In this regard, more precise yet valid methods of elucidating circRNA functions are highly required. However, some questions are highly intriguing and still need further research to be answered satisfactorily. (i) Can miRNA binding efficiently degrade circRNA and affect its half-life? (ii) How significant is the influence of miRNA on circRNA and vice versa? Since miRNA driven circRNA cleavage requires high sequence complementarity between the two, the number of binding sites and circRNA structure may determine the extent of circRNA degradation upon miRNA binding, and thus its half-life. Therefore, it would be interesting to study as to what extent can this regulatory pattern between circRNA and miRNA affect gene expression? These concerns add an additional level of complexity in circRNA-miRNA target-based interaction complexes. Therefore, more analyses are required to be performed experimentally as well as *in silico* to support this idea of interactive networking between circRNA and miRNA.

Apart from the interactions based on the number of binding sites, efficiency to sponge with the target miRNA is also influenced by the tertiary structure of a circRNA. Extensive computational analyses have revealed sequence complementarity to be of utmost importance in regard to circRNA binding with the miRNAs, therefore, determining the stability of the interaction, thus formed. Owing to distinctive tertiary structure formed by the circRNAs, their ability to sponge with the target miRNA can actually be more intricate than what has been described earlier. A circRNA may display various tertiary structures in a cell/tissue specific manner. Within a tertiary structure, these circRNAs may appear to be consisting of a stem loop at one point and/or an imperfectly paired RNA duplex at another, whereby, the binding sites present in the circRNA can exhibit highly diverse sequences with varying complementary. Likewise, single stranded fragment of circRNA may facilitate miRNA sponging to a larger degree as compared to the segment which is already double stranded. Therefore, in order to discern miRNA sponging as a capable mechanism for circRNA function, it is critical to elucidate as to (i) Which part of the circRNA tertiary structure does a complementary miRNA sequence specifically bind to? (ii) How and to what extent does a circRNA tertiary structure affect circRNA-miRNA interaction? (iii) If this complementary binding between miRNA-circRNA forms a stable interaction? (iv) Does circRNA sponging with miRNA explicitly rely on number of binding sites, circRNA tertiary structure, or both? (v) Can a miRNA have multiple varying complementary sequences in the binding circRNA partner? (vi) Can circRNA junctions serve as potential regions of complementary miRNA binding? and (vii) How enriched are circRNAs for miRNA binding sites and vice versa? In order to address these concerns, identification of the circRNA fragment with the highest sequence complementarity for its miRNA binding partner is an aspect that necessitates further research in the field of circRNA biology. Regarding this, we propose the use of RNA pull down technology to bring the circRNA down and the miRNAs that can potentially bind to that circRNA. Nevertheless, this may also pull down various other miRNAs with partial or incomplete complementarity to the circRNA. Therefore, use of adequate controls may help optimize the experimental procedures in a way that enhances the specificity of the RNA-pull down assay and makes circRNA 3D structural dynamics apprehensible.

### 5.2. Hippo-Related circRNAs and Other Regulatory Functions

CircRNAs can perform different regulatory functions depending on their presence in the cytoplasm or nucleus. Nuclear circRNAs can play roles in controlling the first part of the central dogma i.e., transcription, while cytoplasmic circRNAs can regulate its second component i.e., translation and post-translation. The following subsections discuss the importance of circRNAs related to Hippo-YAP axis in regulating transcription, translocation, and translation, while highlighting relevant considerations and outstanding questions.

#### 5.2.1. As Protein Binding Partners

Besides miRNA sponging, circRNAs can also efficiently bind to RNA Binding Proteins (RBPs). This binding may not only halt protein function but also induce conformational changes in protein structure, thus modulating their activities. Our lab previously reported the capability of circFOXO3 to bind to p21 and CDK2 proteins, forming a ternary structure that retards cell cycle progression 159. In general, there is scarcity in the number of studies that endorse this mechanism of circRNA function in various pathological states, particularly in cancer. Further investigation is needed to determine whether other Hippo related circRNAs act as protein binding partners. Furthermore, some of the circRNA complexes with proteins might be too large to allow entry into the cell nucleus or any other organelles in the cell. Now the question is what effect do these circRNAs actually produce, either facilitating the nuclear transportation of proteins or increasing their cytoplasmic retention. In particular, are there any circRNAs that can interact with YAP protein in the cytoplasm, thus affecting its entry into the nucleus? If so, these circRNAs can be potentially used as therapeutic agents to prevent the expression of YAP target genes in cancer. In-depth research studies are needed to resolve this dilemma. In regard to circRNA-protein interactions, most of the studies have highlighted the importance of circRNA sequence, however, there is a negligible count of studies that discuss the structural dynamics of circRNAs in forming circRNA-protein complexes, and their subsequent cellular localization. Therefore, it is imperative to take both circRNA sequence and circRNA structure into account to be better able to apprehend the biological behavior of the circRNAs and the circRNA-protein complexes, thus formed.

#### 5.2.2. As Transcriptional Regulators

Circular RNAs have been demonstrated to regulate both transcription and translation. Unlike other non-coding RNAs, these circularized transcripts can usually exert their transcriptional effects at cis-domain 160. Although circRNAs are mostly cytosolic, an integral subset of circRNAs is found in the nucleus, where they regulate RNA polymerase II driven transcription. However, if Hippo related circRNAs can exert their effects through transcriptional regulation in cancer, this is a domain that requires profound research. Having observed that most of the circRNAs reviewed in this paper exert their functions by targeting YAP1 in cancer, this highlights potential roles for these circRNAs in transcriptional control as well. circAmotl1, a circRNA associated with the Hippo signaling pathway, has been reported by our group to elevate expression of STAT3 and its subsequent translocation, thus regulating the expression of genes associated with mitosis in wound healing 161. Similarly, we also explored the expressional dysregulation of c-Myc as well as its nuclear translocation by circAmotl1 162. There are some intriguing questions that need to be addressed: (i) Are these Hippo-associated circRNAs capable of regulating their host gene transcription or of any other genes? (ii) How efficient are these circRNAs in regulating transcriptional as well as post-transcriptional effects through mRNA trapping? (iii) Do these circRNAs directly bind to any of the components of the pre-initiation complex to modulate transcription? (iv) Since several non-coding RNAs have been reported to be regulating transcription at various levels, is there any interplay of these non-coding RNAs with circRNAs in bringing about transcriptional regulation? (v) Can circRNAs communicate with other forms of non-coding RNAs to drive diverse biological functions in a Hippo-YAP dependent manner? (vi) Can locked nucleic acids (LNA) be helpful in mitigating the effects of oncogenic circRNAs? (vii) If yes, does this cross-talk have a role in altering parental gene transcription for the respective circRNA? (viii) Which steps of the transcription process are actively modulated by these circRNAs? (ix) Do these circRNAs impart any function in RNA Polymerase II phosphorylation during elongation? (x) Can these circRNAs possibly regulate their own expression through post-transcriptional binding with RBPs? (xi) What possible effect can RBPs have on circRNA half-life? The formation of circRNA-protein complexes may increase stability of the circRNAs. Relevantly, our group has reported the binding of circYAP to its parent mRNA, thus inhibiting YAP translation initiation. Nonetheless, it would be helpful to study if this phenomenon has any effect on parent gene transcription. More research studies are needed to unveil any other Hippo relevant circRNAs that can function to regulate transcription factors and to answer the aforementioned concerns in cancer and at large.

#### 5.2.3. As Modulators of Protein Translocation

YAP/TAZ nucleo-cytoplasmic shuttling is a crucial event in Hippo pathway activity. Since circRNAs have been reported to regulate protein translocation, they could also be implicated in modulating YAP/TAZ function serving as an intermediary for nucleus and cytoplasm in tumorigenesis. Generally, nuclear transportation is carried out by importins and requires a protein to have a NLS (Nuclear Localization Signal). Thus, the question arises if there are any circRNAs that possess the potential to regulate YAP/TAZ nucleo-cytoplasmic shuttling? If yes, (i) What underlying mechanisms do these circRNAs follow that allow them to interact with the protein and bring about its translocation? (ii) Does circRNA mediated YAP/YAZ nuclear translocation occur through importins as well? and (iii) Does this circRNA arbitrated shuttling of YAP into the nucleus require the NLS label? These are some of the research questions that need to be rigorously investigated in future studies.

#### 5.2.4. Circular RNA Translation into Proteins

Until recently, circRNAs were always considered to be non-coding molecular entities. During the course of last few years, however, studies have indicated the potential of circRNAs to be effectively translated into distinguishable peptides 163. The proposed mechanisms for circRNA translation are Internal Ribosome Entry Site (IRES)-or N^6^-methyladenosines (m^6^A)-mediated cap-independent translation 164-167. Some outstanding questions regarding their translation are the following: (i) What are the factors that determine the ability of these circRNAs to potentially act as a template for translation? (ii) Can circularization increase RNA efficiency to be translated, since circularization increases circRNA stability? (iii) Is circRNA translation specific to exonic sequences only or those containing both introns and exons can be translated with equal efficiency? (iv) What is the possible mechanism of circRNA translation initiation? (v) What are the factors that determine the fate of circRNA to be translated into a peptide/protein or not? (vi) Does there exist a human circRNA proteome that stayed concealed until now? (vii) Do these circRNA encoded peptides function in harmony with their respective transcripts or incur bifunctional characteristics to the circRNA? Concerning their translation into peptides, low translation initiation efficiency of these circRNAs and lack of protein detection technologies *in-vivo* are the major limitations discerned so far. Moreover, there are only nominal reports to have profiled circRNA translation into peptides under natural conditions. These questions, if answered adequately, would assist in understanding the translation of circRNAs and their protein functions within the cell in a more comprehensive manner.

### 5.3. Feedback Regulation of circRNAs by the Hippo Signaling Pathway

Thus far, the review paper has primarily emphasized the regulation of the Hippo pathway by associated circRNAs in driving tumorigenesis through intricate molecular mechanisms. Meanwhile, if there is any potential mechanism of feedback regulation of these circRNAs or any other endogenous circRNAs by the Hippo signaling pathway is a niche that still remains to be elucidated in cancers and in general. Mechanistic implications of the Hippo pathway components in regulating circRNA expression and function in disease development and progression are discussed in the following section:

#### 5.3.1. Regulation of Splicing Events and circRNA Biogenesis

Initially, circRNAs were considered as by-products of erroneous splicing and hardly caught the due scientific attention until the advancement of RNA sequencing and computational technologies. Currently, they are no longer considered as mere spliced out intronic sequences but can contain exonic sequences as well. In fact, some circRNAs are abundantly expressed. Since circRNAs exhibit cell- and tissue-specific expression 141, it is imperative to examine their expression patterns in different types of cancer. Where circRNAs can be generated as a result of self-splicing (e.g., most of the intronic circRNAs), spliceosome-mediated formation of circRNAs is the most prevalent and highly conserved mechanism of circRNA formation in humans 86. Considering the Hippo signaling pathway, there is ample evidence of the involvement of alternatively spliced Hippo pathway components in disease development and progression 168. Furthermore, YAP1 and TEAD1 have been reported to regulate the splicing of KIBRA (an upstream Hippo pathway signaling molecule) in a feedback manner. Likewise, if the Hippo pathway has the potential to regulate the splicing events that cause the formation of intronic/exonic/intronic-exonic circRNAs in humans, that would be interesting to investigate. To our surprise, no published information was retrieved that explored the role of the Hippo pathway in the back splicing processes, thus regulating circRNA biogenesis. Given the lack of research in the field of circRNA biology, there are many queries that could be addressed: (i) Do Hippo pathway members interact with any of the splicing members or cis-elements that bring about formation of circRNAs? (ii) Can back-splicing provide an additional layer of regulation for Hippo pathway and circRNAs to have diverse roles in different types of cancers? (iii) If yes, what are the possible mechanisms that initiate and sustain such interactions? (iv) Would differently spliced circRNA variants have different regulatory effects on the Hippo pathway and vice versa? (v) Knowing that back-splicing generally requires complementary sequences, how do some genes still opt to form circRNAs otherwise? For example, biogenesis of circMbl is rather dependent on MBL levels and the number of MBL binding sites 169. Hence, it is critical to uncover if circRNA biogenesis can be facilitated by protein factors alone that bind to pre-mRNA, thus bridging the flanking introns together or does it necessarily require complementary sequences to process the formation of circRNAs? (vi) Can Splice-Switching Oligonucleotides (SSOs) affect back-splicing repertoire of the circRNA transcripts? Since these short antisense modified nucleic acids have been reported to alter canonical splicing repertoire by interfering with RNA-RNA binding and/or RNA-Protein interactions 170, it would be equally important to explore if these SSOs have the capacity to affect circRNA repertoire by blocking RNA- protein interactions between pre-mRNA and various components of the splicing machinery during back-splicing or if these modified SSOs do so without altering circRNA abundance. Either way, these chemically modified synthetic SSOs may offer extensive usefulness as therapeutic modulators in future. (vii) How efficiently can circRNAs bind to the splicing factors? (viii) Can binding of one circRNA to the splicing factor facilitate the biogenesis of another circRNA with similar or varying function? (ix) Can the efficiency of back-splicing dictate the fate of circRNAs to be translated into proteins? Because cis elements involved in regulating back splicing have also been known to function as enhancers or repressors of exonic splicing 171, hence, controlling the inclusion of exons to generate either a longer isoform or a shorter one may, subsequently, determine the ability of the circRNA to be translated into a peptide or a protein. (x) Are there any specific splicing factors that can have distinctive effects on the genesis of circRNA? (xi) If yes, what are the expression levels of these factors in pathological states including cancer? (xii) Does the splicing machinery prefer to use intronic sequences for circRNA formation over exonic ones to modulate gene expression? (xiii) Can any of the member of the Hippo pathway potentially disrupt such circRNA-splicing factors interactions? (xiv) If yes, can such complexes, later be exploited as potential hotspots for cancer management? Therefore, we propose these aspects to be of foremost focus in order to understand the role of Hippo pathway molecules in the generation of circRNA transcripts by modulating back-splicing.

#### 5.3.2. Regulation of circRNA Transcription and Translation

It is through the transcriptional loop that the Hippo pathway exerts its functional activities 172. As yet, there is no report that accentuates the feedback regulation of circRNAs by the Hippo pathway components or its target genes. Therefore, lack of relevant findings raises several important questions that should be explored: (i) Can YAP/TAZ hyper-activation mediate the transcription of the circular transcripts from their respective host genes? (ii) Are there any Hippo pathway target genes that can have potential functions in regulating circRNA transcription and translation? (iii) Can these target genes, by any means, modulate the activity of circRNA processing proteins in cancer? (iv) Since YAP/TAZ are considered as important regulators of EMT, are there any EMT drivers that function to enhance transcription and/or translation of oncogenic circRNAs in cancer regulating YAP/TAZ pathway? (v) What possibly can be the cellular consequences of circRNAs expressional regulation by the Hippo signaling in cancer? (vi) Can any of the Hippo pathway genes bind to and function as circRNA interactors to interfere with protein synthesis in cancer? (vii) How are the circRNAs associated with LATS1/2, MST1/2, and YAP/TAZ/TEAD complex regulated in physiological as well as pathological states? (viii) Do Hippo associated circRNA encoded proteins function any differently than those produced by their linear counterparts? (ix) Can Hippo signaling modulate circRNA-protein interactions by interfering with the formation of circRNA tertiary structure? A huge amount of work is required to develop a thorough understanding behind this feedback regulatory mechanism between the Hippo signaling pathway and related circRNAs in cancer.

## 6. Concluding Remarks and Future Prospects

Although there has been convincing evidence on the modulation of the Hippo signaling pathway by these circRNAs in cancer, nevertheless, expanded research is still necessitated to be carried out on the role of circRNAs in the Hippo pathway regulation. Moreover, there are a number of concerns that must be addressed in future studies. Firstly, additional research is required to be carried out to unveil novel circRNAs associated with the Hippo-YAP signaling pathway in cancer. Thus far, few studies have examined the interplay between circRNAs and the Hippo signaling pathway. Secondly, more work is needed to address the functional mechanisms of circRNAs in various forms of cancer.

Elucidation of functional mechanisms of the host gene in conjunction with their circRNAs is lacking in specific forms of cancer. Therefore, cell/ tissue specificity of both the circRNA and its host gene must also be assessed in parallel to describe any possible function in specific cancer types. Furthermore, the Hippo signaling pathway is an extremely important developmental pathway associated with organogenesis and tissue homeostasis. Thus, any circRNA associated with this vital pathway may hold varying functions during different stages of organ development or cancer progression. Therefore, long term studies involving the assessment of circRNA expression and its subsequent function at different time points should be done to acquire insight into circRNA activity during cancer progression. Additionally, the regulatory feedback loops between the upstream signals/ downstream Hippo pathway components and the related circRNAs also require investigation.

Moreover, YAP/TAZ have also been shown to induce epigenetic modifications and regulate chromatin remodeling by interacting with various proteins, thus altering chromatic structure in different pathological states, including cancer. Knowing that circRNAs are the epigenetic signatures associated with cancer progression, it would be interesting to study their possible interactions with YAP/TAZ in bringing about or mitigating their chromatin remodeling effects in cancer. Also, circRNAs have been reported to be methylated. It would, therefore, be intriguing to study the epigenetics status of these circRNAs associated with the Hippo signaling pathway and uncover their mechanistic potential in driving carcinogenesis in humans.

Given that circRNAs are readily detectable in biological specimens from cancer patients, they have the potential to be used as prognostic/ diagnostic markers and therapeutic targets or agents in cancer. However, off-targeting is a limitation of using circRNAs as therapeutic modulators in cancer, as a particular circRNA might be therapeutic in one tissue type while pathogenic in the other.

Moreover, there are several studies that assess the functions of various Hippo pathway related circRNAs in other forms of diseases. Investigating the role of these circRNAs in different types of cancer may also uncover a novel mechanism for circRNA function in tumorigenesis. There are several circRNAs that have been widely implicated in cancer progression, however, these have not been studied in regards to their possible interplay with the Hippo signaling pathway. Discovering these molecular cross-talks and bringing new insights into circRNA-mediated Hippo pathway regulation or Hippo signaling modulation of circRNA function can help in understanding the role of these circRNAs from structural as well as functional perspectives.

As of now, there are quite a few inhibitors/ therapeutic modulators available that target components of the Hippo signaling pathway, nonetheless, if the circRNAs associated with the pathway can be exploited as a subtle therapeutic approach in combating cancer, still requires lot of research and *in vivo* validation through translational studies. Considering the regulatory potential of circRNAs, it would be imperative to use these circRNAs as therapeutic modulators. We speculate that circRNAs that act through miRNA sponging might not be efficacious in therapeutic management of cancer owing to their off-targeting effects. However, the circRNAs that follow protein binding mechanism for their activity or act as translational regulators may serve as potential targets for controlling protein expression and function. Additionally, the circRNAs that play role as transcriptional regulators, and are associated with the gene promoter region can be thought of as the most reasonable targets due to their ability to bind to mRNA directly. Furthermore, we presume that using small oligonucleotides that target and bind to circRNA is relatively more appropriate and less complicated than discovering a whole new molecule that retards protein function.

## Figures and Tables

**Table 1:** CircRNAs associated with the Hippo-YAP pathway and their functional implications

Cancer Type	CircBase ID	Expression (up/down)	Gene Function	Mechanism	Pathway	Sample(s) Used	Sample Size	Study Method/ Technique	Clinical Application	Ref
breast, liver, melanoma	circYAP	up-regulated	tumor suppressor	inhibiting Yap translation	YAP1	cells	-	WB, RIP, RNA pull down	cancer intervention	80
cervical	hsa_circ_0023404	up-regulated	oncogene	miR-136 sponge	YAP1	tissue	n = 53 pairs	qPCR, siRNA silencing	prognosis marker	72
colon	circPPP1R12A/hsa_circ_0000423	up-regulated	oncogene	activating Hippo-YAP pathway	YAP1	tissue, cells	n = 20 pairs	circRNA-array, qPCR, FISH, Nuclear Mass Separation Assay, LC/MS, RNA-seq	prognosis marker	73
hepatocellular carcinoma	circRNA_104075	up-regulated	oncogene	miR-582-3p sponge	YAP1	tissues, cells and serum	healthy control n = 60; tissue pairs n = 10	CRISPR/Cas9, ChIP, RNA-IP, circRNA probe precipitation, ELISA	diagnostic marker, therapeutic target	74
multiple myeloma	circCDYL	up-regulated	oncogene	miR-1180 sponge	YAP1	bone marrow, peripheral blood, cells	patients n = 72 and healthy donors n = 13	qPCR, WB, Luc-assay, RNA pull down, Xenograft tumor model	therapeutic target	75
osteosarcoma	circFAT1	up-regulated	oncogene	miR-375 sponge	YAP1	tissues, cells and mouse model	osteosarcoma n = 12, chondroma n = 12	WB, qRT-PCR, FISH, chromogenic in situ hybridization, RNA binding protein immunoprecipitation and immunofluorescence, xenograft,	therapeutic target	78
head and neck squamous carcinoma	circPVT1	up-regulated	oncogene	miR-497-5p sponge	YAP/TEAD Complex	tissues, cells	n = 115 pairs	qPCR, WB, ChIP, RNA immunoprecipitation, DRB-4sU assay, In-Silico prediction analysis,	diagnostic, therapeutic target	81
colorectal	circ0106714	down-regulated	tumor suppressor	miR-942-5p sponge	YAP1	tissues, cells, mouse models	n = 60 pairs	RNase R assay, qPCR, WB, RNA-IP, flowcytometric and apoptotic assay, Luc-assay, xenograft, immunohistochemistry, H&E	prognosis, therapy of CRC.	79
cholangiocarcinoma	circRNA_000585	up-regulated	oncogene	miR-615-5p sponge	AMOT, YAP1	CCA and para-cancer tissue	n = 15	microarray hybridization, qPCR, bioinformatic analysis	therapeutic Target	76
gastric	circLARP4	down-regulated	tumor suppressor	miR-424-5p sponge	LATS1	TCGA RNA-sequencing, cells	387 Cases	dual Luc-assay and RIP assay, FISH	prognostic Marker	64
oral squamous cell carcinoma	circRNA_0000140	down-regulated	tumor suppressor	miR-31 sponge	LATS2	tissue, cells, xenograft	n = 56 pairs	Sanger-seq, q-PCR, WB, immunofluorescence, and immunohistochemistry, Luc-assay and Argonaute 2-RIP assays	diagnostic, prognosis marker	65
breast (TNBC)	hsa_circ_0091074	up-regulated	oncogene	miR‑1297e sponge	TAZ	cells	-	qPCR, WB, functional assays, dual-Luc-assay,	therapeutic target for TNBC	82
Breast	hsa_circ_0005273	up-regulated	oncogene	sponging miR-200a-3p	YAP1	tissues, cells, xenograft		qPCR, RIP assay, RNA probe pull-down assay, luciferase reporter assay and FISH	biomarker, therapeutic target	77
Breast	hsa_circ_0001667	up-regulated	oncogene	miR-125a-5p sponge	TAZ	tissues, cells, bioinformatics		qPCR, WB, Functional Assays, dual-Luc-assay	biomarker, therapeutic target	83

**Table 2:** Prognostic and therapeutic potential of circRNAs associated with the regulatory (inhibitory) module of the Hippo pathway

circRNA Symbol/ID	Cancer Type	circRNA Function	Associated Pathway Component	Prognostic/ Diagnostic Potential	Therapeutic Potential	Reference
circRNA_000585	Cholangiosarcoma	Oncogene	AMOT	-	+	76
circLARP4	Gastric Cancer	Tumor Suppressor	LATS1	+	-	64
circRNA_0000140	Oral Squamous Cell Carcinoma	Tumor Suppressor	LATS2	+	-	65

**Table 3:** Prognostic and therapeutic potential of circRNAs associated with the transcriptional module of the Hippo Pathway

circRNA Symbol/ID	Cancer Type	circRNA Function	Associated Pathway Component	Prognostic/ Diagnostic Potential	Therapeutic Potential	Reference
circYAP	Breast Cancer, Liver Cancer	Tumor Suppressor	YAP	-	+	80
hsa_circ_0023404	Cervical Cancer	Tumor Suppressor	YAP	+	-	72
circPPP1R12A/ hsa_circ_0000423	Colon Cancer	Oncogene	YAP	+	-	106
circRNA_104075	Hepatocellular Carcinoma	Oncogene	YAP	+	+	74
circCDYL	Multiple Myeloma	Oncogene	YAP	-	+	75
circFAT1	Osteosarcoma	Oncogene	YAP	-	+	78
circ0106714	Colorectal Cancer	Tumor Suppressor	YAP	+	+	79
circRNA_000585	Cholangiosarcoma	Oncogene	YAP	-	+	76
hsa_circ_0005273	Breast Cancer	Oncogene	YAP	+	+	76
hsa_circ_0091074	Breast Cancer	Oncogene	TAZ	-	+	82
circ_0001667	Breast Cancer	Oncogene	TAZ	+	+	82
circPVT1	Head and Neck Squamous Cell Carcinoma	Oncogene	YAP/TEAD	+	+	81

**Table 4:** Hippo pathway associated circRNAs as therapeutic modulators in cancer

*circRNA*	*Molecular Target*	*Therapeutic Function*	*Stage of Clinical Testing*	*References*
*circYAP*	YAP1	Therapeutic Agent	In vitro, In vivo	80
*hsa_circ_0023404*	YAP1	Therapeutic Target	In vitro	72
*hsa_circ_0000423*	YAP1	Therapeutic Target	In vitro	73
*circRNA_104075*	YAP1	Therapeutic Target	In vitro	74
*circCDYL*	YAP1	Therapeutic Target	In vitro	75
*circFAT1*	YAP1	Therapeutic Target	In vitro	78
*circPVT1*	YAP1/TEAD	Therapeutic Target	In vitro	81
*circ0106714*	YAP1	Therapeutic Agent	In vitro, In vivo	79
*circRNA_000585*	AMOT, YAP1	Therapeutic Target	In vitro	76
*circLARP4*	LATS1	Therapeutic Agent	In vitro	64
*circRNA_0000140*	LATS2	Therapeutic Agent	In vitro, In vivo	65
*hsa_circ_0091074*	TAZ	Therapeutic Target	In vitro	82
*hsa_circ_0005273*	YAP1	Therapeutic Target	In vitro, In vivo	77
*hsa_circ_0001667*	TAZ	Therapeutic Target	In vitro	83

## Abbreviations

circRNA: circular RNA
pre-mRNAs: precursor mRNAs
miRNA: microRNA
siRNA: interference RNA
YAP: Yes associated protein
TAZ: transcriptional co-activator
LATS1/LATS2: Large Tumor Suppressor 1 and 2
SAV1: Salvador homolog 1
SSO: Splice-Switching Oligonucleotide
RBP: RNA-binding protein
IRESs: Internal ribosome entry sites
ORF: open reading frame
PKR: protein kinase
